# Effects of Resvega on Inflammasome Activation in Conjunction with Dysfunctional Intracellular Clearance in Retinal Pigment Epithelial (RPE) Cells

**DOI:** 10.3390/antiox10010067

**Published:** 2021-01-07

**Authors:** Niina Bhattarai, Niina Piippo, Sofia Ranta-aho, Yashavanthi Mysore, Kai Kaarniranta, Anu Kauppinen

**Affiliations:** 1 School of Pharmacy, Faculty of Health Sciences, University of Eastern Finland, 70210 Kuopio, Finland; niina.piippo@uef.fi (N.P.); sofia.ranta-aho@uef.fi (S.R.-a.); yashavanthi.mysore@uef.fi (Y.M.); 2 Department of Ophthalmology, Institute of Clinical Medicine, University of Eastern Finland, 70210 Kuopio, Finland; kai.kaarniranta@uef.fi; 3 Department of Ophthalmology, Kuopio University Hospital, 70210 Kuopio, Finland

**Keywords:** Resvega, resveratrol, antioxidant, NLRP3 inflammasome, caspase-1, IL-1β, ARPE-19 cell, RPE cell, autophagy

## Abstract

Age-related macular degeneration (AMD) is an eye disease in which retinal pigment epithelium (RPE) cells play a crucial role in maintaining retinal homeostasis and photoreceptors’ functionality. During disease progression, there is increased inflammation with nucleotide-binding domain, leucine-rich repeat, and Pyrin domain 3 (NLRP3) inflammasome activation, oxidative stress, and impaired autophagy in RPE cells. Previously, we have shown that the dietary supplement Resvega reduces reactive oxygen species (ROS) production and induces autophagy in RPE cells. Here, we investigated the ability of Resvega to prevent NLRP3 inflammasome activation with impaired protein clearance in human RPE cells. Cell viability was measured using the lactate dehydrogenase (LDH) and the 3-(4,5-dimethylthiazol-2-yl)-2,5-diphenyltetrazolium bromide (MTT) assays. Enzyme-linked immunosorbent assays (ELISA) were utilized to determine the secretion of cytokines, NLRP3, and vascular endothelial growth factor (VEGF). Caspase-1 activity was measured with a fluorescent labeled inhibitor of caspase-1 (FLICA; FAM-YVAD-FMK) and detected microscopically. Resvega improved the cell membrane integrity, which was evident as reduced LDH leakage from cells. In addition, the caspase-1 activity and NLRP3 release were reduced, as was the secretion of two inflammatory cytokines, interleukin (IL)-1β and IL-8, in IL-1α-primed ARPE-19 cells. According to our results, Resvega can potentially reduce NLRP3 inflammasome-mediated inflammation in RPE cells with impaired protein clearance.

## 1. Introduction

Age-related macular degeneration (AMD) is an eye disease that leads to severely impaired central vision mainly as a consequence of the degeneration of retinal pigment epithelial (RPE) cells [1]. RPE cells, which are crucial for the maintenance of photoreceptor functionality and retinal homeostasis, are prone to inflammation, increased oxidative stress, and impaired autophagy [1,2]. Generally, AMD is divided into dry and wet forms [1]. The damage of RPE cells is typical for both disease forms but abnormal neovascularization is a clinical hallmark in wet AMD [1]. Chronic inflammation is involved in both disease forms [2]. 

Changes in the reactive oxygen species (ROS) production, nucleotide-binding domain, leucine-rich repeat, and Pyrin domain 3 (NLRP3) inflammasome activation, and autophagy mutually interact with each other [3,4,5,6,7,8]. For example, increased ROS production should trigger autophagy in order to protect the cell from acute oxidative stress [6,9]. Instead, overwhelming ROS production and impaired autophagy can result in inflammasome activation [3,5,7,8,10,11,12,13,14]. On the other hand, functional autophagy is needed to remove inflammasome components after the activation process [5,15,16]. Autophagy-mediated removal of damaged organelles and protein aggregates also reduces ROS production and indirectly suppresses the activation of the inflammasome [1,5,8,13,17]. NLRP3 expression has been detected in the eyes of patients suffering from either dry or wet AMD but not in those of unaffected control subjects [18].

NLRP3 belongs to the cytosolic NLR (nucleotide-binding domain, leucine-rich repeat) protein receptors that recognize danger signals and contribute to the pathogenesis underpinning AMD [1,2,18,19]. NLRP3 activation leads to the oligomerization of the NLRP3 inflammasome complex, in which apoptosis-associated speck-like protein containing a caspase-recruitment domain (ASC) links NLRP3 with pro-caspase-1, resulting in the activation of caspase-1 and the cleavage of pro-interleukin (IL)-1β and/or pro-IL-18 into their mature secreted forms [2,12,20,21]. Pro-IL-1β expression is absent under normal conditions and requires a priming signal mediated through a Toll-like receptor (TLR) or a cytokine receptor [18,22]. Pathogen or damage-associated molecular patterns (PAMPs or DAMPs) represent activation signals to NLRP3. In AMD, many factors, such as ROS production, lysosomal destabilization, purinergic receptor X7 (P2X7) activation, increased extracellular adenosine triphosphate (ATP), K^+^-efflux with Ca^2+^-influx are all able to induce NLRP3 activation [2,3,5,8,11,18,23,24,25,26]. We have previously shown that the disrupted protein clearance induced by proteasome (MG-132; MG) and autophagy (bafilomycin A1; BafA) inhibitors results in the activation of the NLRP3 inflammasome and IL-1β secretion through increased ROS production in ARPE-19 cells [4,10].

Resvega (Laboratoires Théa, Clermont-Ferrand, France) is a mixture including omega-3 fatty acids (eicosapentaenoic acid (EPA) 30% *w/w*; docosahexaenoic acid (DHA) 15% *w/w*; docosapentaenoic acid (DPA) 4% *w/w*), vitamins C (19% *w/w*) and E (2% *w/w*), trans-resveratrol (2% *w/w*), lutein (1% *w/w*), zeaxanthin (0.2% *w/w*), copper (0.1% *w/w*), and zinc (1% *w/w*). In comparison to the Age-Related Eye Disease Study (AREDS) formulations, Resvega has resveratrol as an additional component [27,28]. In previous studies on ARPE-19 cells, Resvega has reduced hydroquinone-mediated ROS production and promoted autophagy during impaired protein clearance [29,30]. Resveratrol alone has been shown to induce autophagy and evoke anti-inflammatory responses in RPE cells as well inhibit NLRP3 expression, caspase-1 activation, and IL-1β secretion in a mouse model of acute lung injury [31,32].

In this study, we investigated the ability of Resvega to alleviate NLRP3 inflammasome activation in human RPE cells with disrupted protein clearance since this plays a major role in the pathogenesis of AMD [18]. Resvega reduced IL-1β and NLRP3 secretion concurrently with reduced caspase-1 activity, evidence that it may potentially alleviate NLRP3 inflammasome activation.

## 2. Materials and Methods 

### 2.1. Cell Culture and Treatments 

The experiments were conducted using the ARPE-19 cell line (ATCC). Cells were cultured in Dulbecco’s modified Eagle’s medium (DMEM) with nutrient mixture F-12 (1:1) (Life Technologies, Paisley, UK) with the addition of 2 mM L-glutamine (Life Technologies, Paisley, UK), 100 U/mL penicillin with 100 μg/mL streptomycin (Life Technologies, Grand Island, NY, USA), and 10% fetal bovine serum (GE Healthcare Life Sciences, South Logan, UT, USA). The cells were seeded onto 12-well plates (Costar, Corning Inc., Kennebunk, ME, USA) at a density of 200,000 cells per well and grown for three days in serum-containing medium at +37 °C, 5% CO_2_. 

The experiments were performed in growth medium without a serum supplement. First, ARPE-19 cells were exposed to different concentrations of Resvega (0.1–100 µM; Laboratoires Théa, Clermont-Ferrand, France) based on the concentrations of resveratrol for 24 h at +37 °C, 5% CO_2_. Resvega concentrations 0.1–25 μM were selected for further investigations. The cells were primed with recombinant human IL-1α (4 ng/mL, R&D Systems, Minneapolis, MN, USA) for 24 h at +37 °C, 5% CO_2_ to induce the expression of NLRP3 and the pro-IL-1β and pro-caspase-1, as described previously [4,16,18]. Thereafter, Resvega, containing omega-3 fatty acids (EPA 30% *w/w*; DHA 15% *w/w*; DPA 4% *w/w*), resveratrol (2% *w/w*), vitamins C (19% *w/w*) and E (2% *w/w*), copper (0.1% *w/w*), zinc (1% *w/w*), lutein (1% *w/w*), and zeaxanthin (0.2% *w/w*), was added 1 h before the proteasome inhibitor MG-132 (MG; 5 μM, Calbiochem, San Diego, CA, USA) and both were left on cell cultures concurrently for another 24 h. Subsequently, the autophagy inhibitor bafilomycin A1 (BafA; 50 nM, Cayman chemicals, Michigan, MI, USA) was added for additional 24 h. Resvega concentrations of 0.1, 1, 10 and 25 μM were included in viability assays, with Resvega 10 and 25 μM being chosen for measurement of cytokines IL-6 and IL-8, and Resvega 10 μM for IL-1β, NLRP3, caspase-1, ATP, and vascular endothelial growth factor (VEGF) measurements. Resvega was diluted by dimethyl sulfoxide (DMSO; 0.5% *v/v*, Medintech Inc., Corning, Manassas, VA, USA) which served as the diluent control to Resvega. Untreated cells served as controls for IL-1α-primed cells, and IL-1α for IL-1α-primed cells with inflammasome activators MG-132 and BafA. After the exposures, half of the medium was collected and centrifuged (Biofuge Fresco Heraeus Instruments, Newport Pagnell, UK) at 381 g for 10 min at +4 °C. The lactate dehydrogenase (LDH) release and the secretion of cytokines, NLRP3, ATP, and VEGF were determined from medium samples. Cells and the rest of the medium were subjected to the thiazolyl blue tetrazolium bromide (MTT) assay. 

In the caspase-1 activity measurement, the cells were seeded onto 8-well plates (Lab-Tek™ Chamber Slide System, Thermo Fisher Scientific, Rochester, NY, USA) at a density of 40,000 cells per well and grown for two days in serum-containing medium at +37 °C, 5% CO_2_. The cells were primed with IL-1α (4 ng/mL) for 24 h at +37 °C, 5% CO_2_. Subsequently, the cells were exposed to 10 μM Resvega for 1 h, followed by a treatment with MG-132 (MG; 5 μM) for another 24 h. Thereafter, bafilomycin A1 (BafA; 50 nM) was added for an additional 6 h and incubated for the last hour concurrently with a fluorescently labeled inhibitor of caspase-1 (FLICA; 1× FAM-YVAD-FMK, ImmunoChemistry Technologies LLC, Bloomington, MN, USA). DMSO (1% *v/v*), in which Resvega was dissolved, serving as the diluent control upon MG-132 and BafA treatments with IL-1α-priming, and IL-1α was used as a control for MG-132 and BafA treatments on IL-1α-primed cells. After all exposures, the cells were washed (2 × 10 min at + 37 °C, 5% CO_2_) using the kit-specific washing buffer, and fixed with 4% paraformaldehyde (PFA) for 15 min at room temperature. Nuclei were stained using the Hoechst 33342 dye (NucBlue Live Cell Stain, Life Technologies, Eugene, OR, USA). After the stainings, the Mowiol (Sigma, St. Louis, MO, USA) mounting medium was added and pictures were taken on the next day using the Zeiss ApoTome.2 Imager M2 microscope with the Zen pro 2012 program (Carl Zeiss Microscopy GmbH, Jena, Germany). 

### 2.2. Cell Viability Assays 

Lactate dehydrogenase (LDH) release was analyzed from medium samples immediately after the treatments using a commercial kit (CytoTox96^®^ Non-Radioactive Cytotoxicity Assay, Promega, Madison, WI, USA). Absorbance values were determined at a wavelength of 490 nm in a spectrophotometer BioTek ELx808 with the Gen-5 2.04 program (BioTek Instruments Inc., Winooski, VT, USA). 

Cellular metabolic activity was determined using the thiazolyl blue tetrazolium bromide (MTT) assay (Sigma-Aldrich, St Louis, MO, USA). After the treatments, 25 μL MTT salt (10 mg/mL) was added to the cells for 3 h at +37 °C, 5% CO_2_. The assay was performed as described previously [29]. Absorbance values were determined using the spectrophotometer at a wavelength of 560 nm.

### 2.3. Enzyme-Linked Immunosorbent Assay (ELISA) 

An enzyme-linked immunosorbent assay (ELISA) was used to measure the amounts of pro-inflammatory cytokines IL-6, IL-8, and IL-1β from medium samples using BD OtpEIA^TM^ Human ELISA Kits (BD Biosciences, San Diego, CA, USA). NLRP3 was assayed in the medium using the Human NACHT, LRR and PYD domains-containing protein 3 (NLRP3/c1orf7/CIAS1/NALP3/PYPAF1) ELISA kit (Cusabio Biotech co., LTD, Wuhan, Hubei Province, China) and VEGF using the DuoSet Human VEGF kit (R&D Systems, Minneapolis, MN, USA). Absorbance values were detected at a wavelength of 450 nm with a correction at 655 nm using a microplate reader Bio-Rad Model 550 with the Microplate Manager 5.2 program (Bio-Rad Laboratories Inc., Hercules, CA, USA).

### 2.4. ATP Measurement

Extracellular ATP levels were measured from medium samples using a commercial kit according to the manufacturer’s instructions (Promega, Madison, WI, USA). The luminescence signal was detected using BioTek Cytation3 imaging reader with Gen-5 3.03 program (Instruments Inc., Winooski, VT, USA). 

### 2.5. Statistical Analyses 

All data were analyzed using the GraphPad Prism program 7.04 (GraphPad Software, San Diego, CA, USA) and pairwise comparisons between study groups were performed using the Mann–Whitney U-test. Results are shown as mean ± standard error of means (SEM). Differences were considered as statistically significant with *p*-values lower than 0.05 (* *p* < 0.05, ** *p* < 0.01, *** *p* < 0.001, **** *p* < 0.0001). 

## 3. Results

### 3.1. Resvega Preserves the Cell Membrane Integrity upon Impaired Protein Clearance

**Figure 1:**
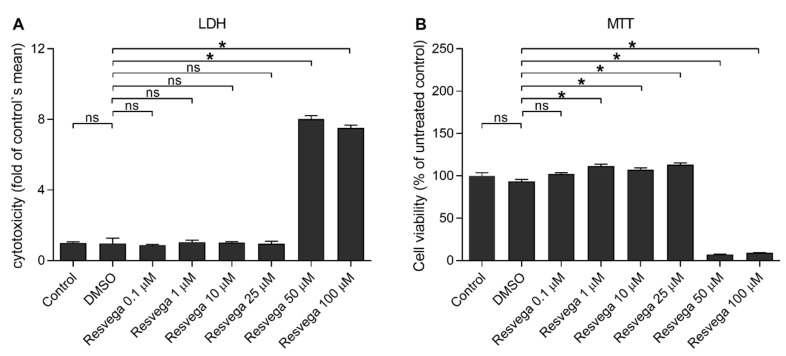
The effect of Resvega (0.1–100 μM; dimethyl sulfoxide (DMSO) 0.5% *v/v*) on the cell viability in ARPE-19 cells. Rupturing of the cell membrane is indicated by the lactate dehydrogenase (LDH) assay (**A**), and metabolic activity using the 3-(4,5-dimethylthiazol-2-yl)-2,5-diphenyltetrazolium bromide (MTT) assay (**B**). Results are compared to the untreated control group, which was set to be 1 (**A**) or 100% (**B**). Resvega was diluted by DMSO which served as the diluent control to Resvega. Data were collected from 1 experiment containing 4 parallel samples per group. Results are shown as mean ± standard error of mean (SEM) and were analyzed using the Mann–Whitney U-test. * *p* < 0.05, ns—not significant.

**Figure 2:**
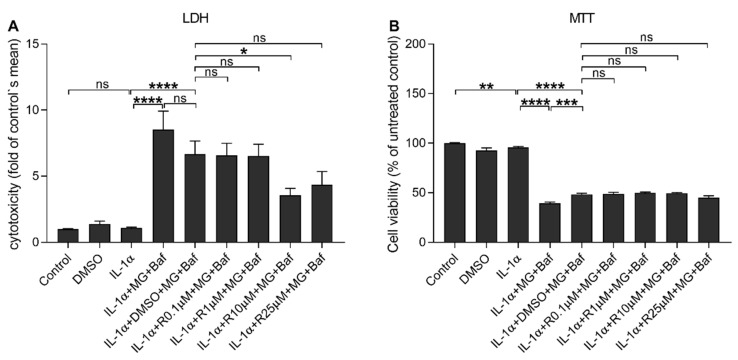
The effect of Resvega (R 0.1–25 μM; DMSO 0.5% *v/v*) on the cell viability in IL-1α-primed ARPE-19 cells in conjunction with proteasome (MG; 5 μM) and autophagy (bafilomycin A1 (BafA); 50 nM) inhibitor treatments. Rupturing of cell membrane is indicated by the LDH assay (**A**) and metabolic activity using the MTT assay (**B**). Results are compared to the untreated control group, which was set to be 1 (**A**) or 100% (**B**). Resvega was diluted by DMSO which served as the diluent control to Resvega. Untreated cells served as controls for interleukin (IL)-1α-primed cells, and IL-1α for IL-1α-primed cells with inflammasome activators, MG-132 and BafA. Data are combined from 3 independent experiments containing 4 parallel samples per group in each experiment. Results are shown as mean ± standard error of mean (SEM) and analyzed using the Mann–Whitney U-test. * *p* < 0.05, ** *p* < 0.01, *** *p* < 0.001, **** *p* < 0.0001, ns—not significant.

First, we investigated the effect of Resvega on the cell viability in ARPE-19 cells using LDH and MTT assays (Figure 1). Resvega was well-tolerated from a concentration of 0.1 to 25 μM but cytotoxicity was shown from a 50 μM concentration of Resvega according to the LDH assay (Figure 1A). A low concentration (0.1 μM) of Resvega had no additional effect on the MTT assay but concentrations of 1–25 μM improved the MTT salt processing (Figure 1B). Metabolic activity declined from 50 μM Resvega concentration. Based on the cell viability assays, Resvega concentrations of 0.1–25 μM were selected for further experiments to study the cell viability of IL-1α-primed ARPE-19 cells with impaired protein clearances induced by the proteasome and autophagy inhibitors MG-132 and bafilomycin A1, respectively [16]. Resvega at 10 μM significantly reduced the lactate dehydrogenase (LDH) release from RPE cells with the disrupted intracellular clearance when compared to cells exposed to MG and BafA with the DMSO solvent (Figure 2A). Furthermore, the lower Resvega concentrations (0.1–1 μM) or the highest concentration (25 μM) had no effect on the LDH release. According to the thiazolyl blue tetrazolium bromide (MTT) assay, the disrupted protein clearance compromised the metabolic activity of ARPE-19 cells, and Resvega had no ability to prevent that phenomenon (Figure 2B). 

### 3.2. Resvega Reduces the IL-8 Secretion from IL-1α-Primed ARPE-19 Cells upon Dysfunctional Cellular Clearance

**Figure 3:**
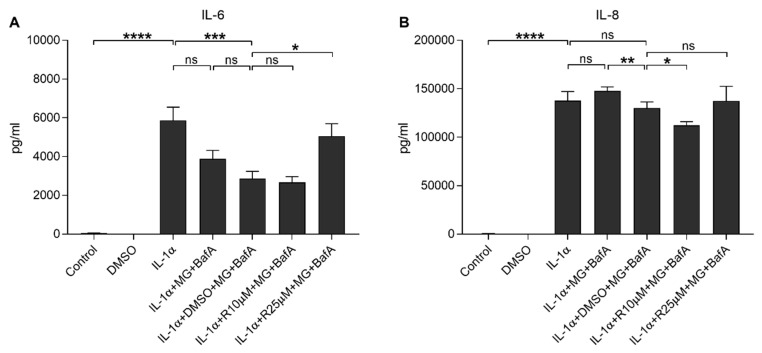
The effect of Resvega (R10–25 μM; DMSO 0.5% *v/v*) on the secretion of inflammatory cytokines IL-6 (**A**) and IL-8 (**B**) upon MG-132 (MG; 5 μM) and bafilomycin A1 (BafA; 50 nM) exposure in IL-1α-primed ARPE-19 cells. Resvega was diluted by DMSO which served as the diluent control to Resvega. Untreated cells served as controls for IL-1α-primed cells, and IL-1α for IL-1α-primed cells with inflammasome activators MG-132 and BafA. Data are combined from 3 independent experiments containing 4 parallel samples per group in each experiment. Results are shown as mean ± SEM and analyzed using the Mann–Whitney U-test. * *p* < 0.05, ** *p* < 0.01, *** *p* < 0.001, **** *p* < 0.0001, ns—not significant.

Next, we studied the effect of Resvega on the production of pro-inflammatory cytokines. IL-1α induced the release of IL-6, which was significantly reduced by MG-132 and BafA treatments with DMSO control (Figure 3A). Similarly, IL-1α induced the secretion of IL-8, which remained unchanged after the addition of MG-132 and BafA (Figure 3B). Resvega (10 µM) exerted no additional effect on the IL-6 secretion but the 25 μM concentration increased IL-6 release when compared to MG-132 and BafA treatments with DMSO (Figure 3A). A lower Resvega concentration (10 µM) significantly reduced the IL-8 secretion (Figure 3B) but it was not visible on cells exposed to 25 μM Resvega when compared to IL-1α-primed ARPE-19 cells exposed to MG-132 and BafA with DMSO. Since 10 μM Resvega maintained the integrity of the cell membrane and reduced the secretion of IL-8 in these ARPE-19 cells, we selected this concentration for further studies. 

### 3.3. Resvega Alleviates the NLRP3 Inflammasome Activation in IL-1α-Primed ARPE-19 Cells Which Have Impaired Protein Clearance

**Figure 4:**
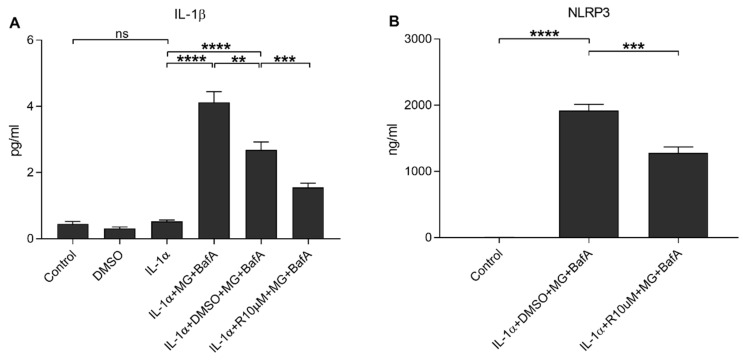
The effect of Resvega (R10 μM; DMSO 0.5% *v/v*) on the secretion of IL-1β (**A**) and NLRP3 (**B**) upon MG-132 (MG; 5 μM) and bafilomycin A1 (BafA; 50 nM) exposure in IL-1α-primed ARPE-19 cells. Resvega was diluted by DMSO which served as the diluent control to Resvega. Untreated cells served as controls for IL-1α-primed cells, and IL-1α for IL-1α-primed cells with inflammasome activators MG-132 and BafA. Data are combined from 3 independent experiments containing 4 (**A**) or 3 (**B**) parallel samples per group in each experiment. Results are shown as mean ± SEM and analyzed using the Mann–Whitney U-test. ** *p* < 0.01, *** *p* < 0.001, **** *p* < 0.0001, ns—not significant.

**Figure 5:**
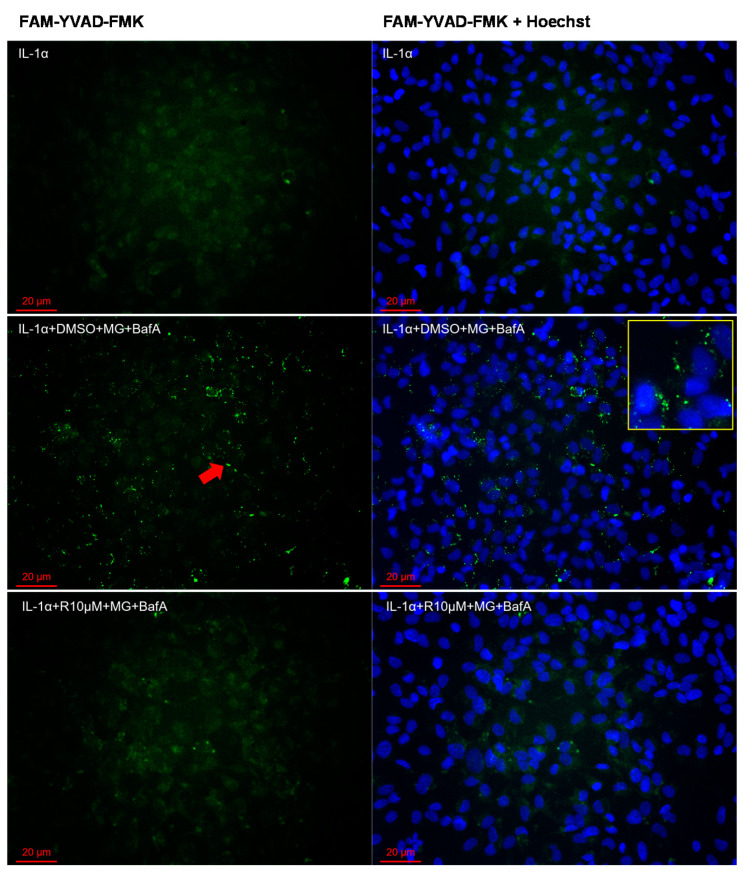
The effect of Resvega (R10 μM; DMSO 1% *v/v*) on the caspase-1 activity in conjunction with MG-132 (MG; 5 μM) and bafilomycin A1 (BafA; 50 nM) exposure in IL-1α-primed ARPE-19 cells. The caspase-1 activity was investigated using a specific fluorochrome inhibitor of caspase-1 (FLICA, FAM-YVAD-FMK) and detected in a fluorescent microscope (Zeiss ApoTome.2 Imager M2 microscope). A green fluorescent signal (pointed as an example by a red arrow) indicates the presence of active caspase-1 attached to the FAM-YVAD-FMK-probe. Nuclei were stained using the blue Hoechst 33342 dye. Resvega was diluted by DMSO which served as the diluent control to Resvega, and IL-1α for IL-1α-primed cells with inflammasome activators MG-132 and BafA. One representative picture is presented from each group (a more extensive combination of pictures is included in the Supplementary Materials; Figures S1–S3).

We have previously shown that dysfunctional protein clearance could be induced by the proteasome and autophagy inhibitors MG-132 and BafA, respectively; this then activates the NLRP3 inflammasome, resulting in the activation of caspase-1 and the subsequent secretion of IL-1β and NLRP3 from human RPE cells [10,16]. In the present study, Resvega reduced the release of IL-1β and NLRP3 in the same cell model (Figure 4). Resvega also reduced the intracellular caspase-1 activity, which was determined using a specific fluorochrome inhibitor of caspase-1 (FLICA, FAM-YVAD-FMK; Figure 5, Figures S1–S3). 

### 3.4. Resvega Increases Extracellular ATP Levels in IL-1α-Primed ARPE-19 Cell Cultures upon Exposure to Proteasome and Autophagy Inhibitors 

**Figure 6:**
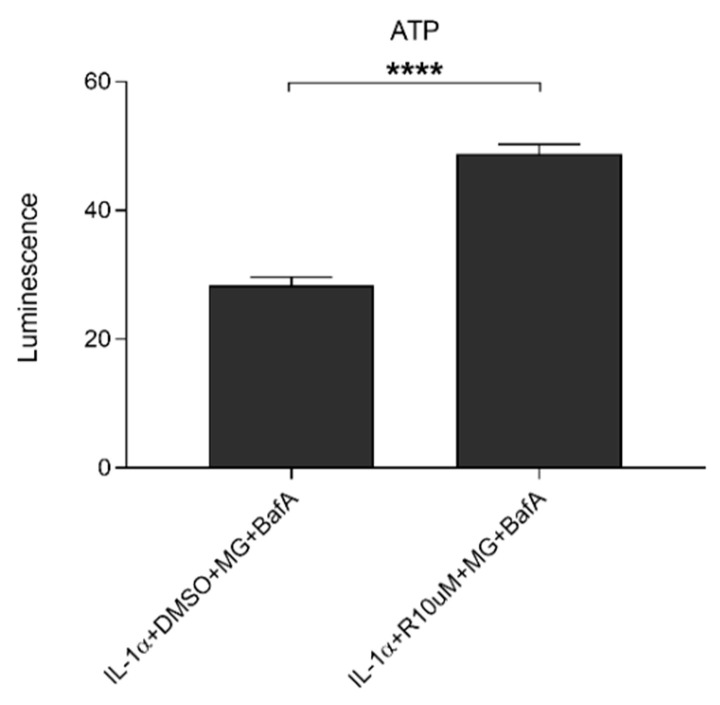
The effect of Resvega (R10 μM; DMSO 0.5% *v/v*) on extracellular ATP levels in the presence of MG-132 (MG; 5 μM) and bafilomycin A1 (BafA; 50 nM) in IL-1α-primed ARPE-19 cells. Resvega was diluted by DMSO which served as the diluent control to Resvega. Data are combined from 3 independent experiments containing 3 parallel samples per group in each experiment. Results are shown as mean ± SEM and analyzed using the Mann–Whitney U-test. **** *p* < 0.0001.

In our previous study, MG-132 and BafA treatments induced the release of ATP from IL-1α-primed ARPE-19 cells [4]; here, we wanted to examine whether Resvega has an effect on this. As seen in Figure 6, Resvega further increased the levels of extracellular ATP from the cells with impaired protein clearance. The increase was statistically significant and 1.7 times higher in the presence of Resvega than without it.

### 3.5. Resvega Reduces IL-1α-Induced VEGF Secretion 

**Figure 7:**
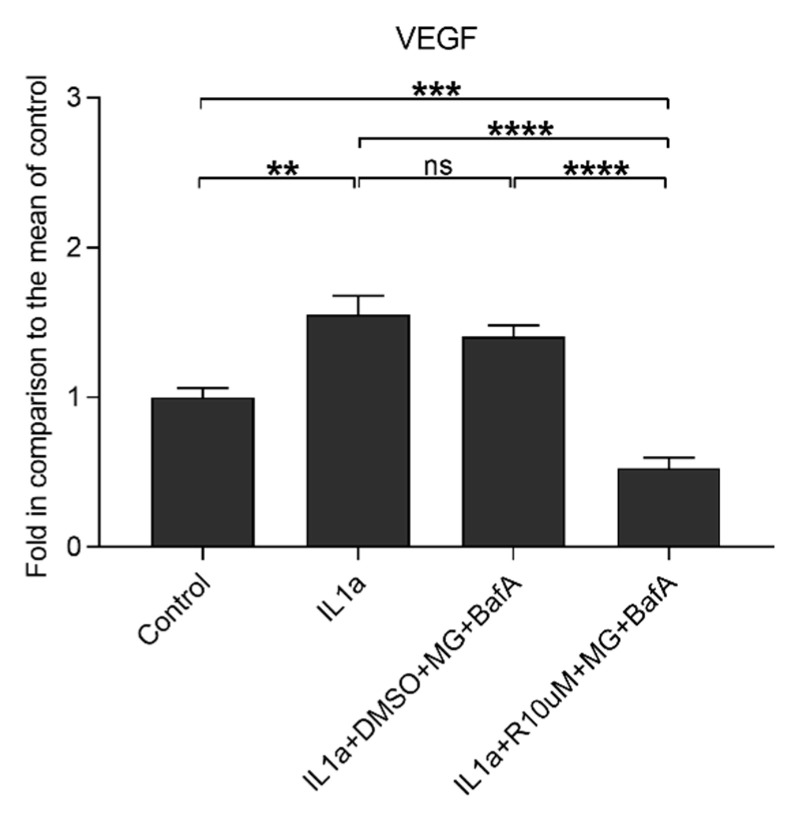
The effect of Resvega (R10 μM; DMSO 0.5% *v/v*) on the vascular endothelial growth factor (VEGF) secretion in IL-1α-primed ARPE-19 cells treated with MG-132 (MG; 5 μM) and bafilomycin A1 (BafA; 50 nM). Results are compared to the untreated control group, which was set to be 1. Resvega was diluted by DMSO which served as the diluent control to Resvega. Untreated cells served as controls for IL-1α-primed cells, and IL-1α for IL-1α-primed cells with inflammasome activators MG-132 and BafA. Data are combined from 3 independent experiments containing 3 parallel samples per group in each experiment. Results are shown as mean ± SEM and analyzed using the Mann–Whitney U-test. ** *p* < 0.01, *** *p* < 0.001, **** *p* < 0.0001, ns—not significant.

We have previously shown that changes in redox balance upon hypoxic conditions induce VEGF secretion in ARPE-19 cells [33]. Our data show that IL-1α significantly increased the release of VEGF and MG-132 (MG) with bafilomycin A1 (BafA) having no additional effect on said property (Figure 7). Resvega significantly reduced VEGF secretion when compared to cells with disrupted cellular clearance without Resvega supplementation. The level to which Resvega reduced VEGF was significantly lower than that detected in untreated cells or in cells exposed to IL-1α alone.

## 4. Discussion

During aging, there is an accumulation of protein aggregates in the retina, which is a major risk factor for AMD [1,34]. The impaired protein clearance in RPE cells results in increased oxidative stress and inflammation through the activation of the NLRP3 inflammasome [1,10,12,16,17]. The nutritional supplement Resvega has many components (omega-3 fatty acids, vitamins C and E, resveratrol, lutein, zeaxanthin, copper, and zinc) that belong to the normal antioxidant defense of the retina [35,36]. In the present study, Resvega was well-tolerated until 25 μM concentration. It is in line with our previous study where we saw that 25 μM Resvega alone did not compromise cell viability [30]. We have previously shown that Resvega can improve cell viability determined by both MTT and LDH assays during hydroquinone-induced oxidative stress or MG-132 exposure on ARPE-19 cells [29,30]. In the present study, Resvega preserved the integrity of cell membranes with disrupted protein clearance but had no effect on the cellular metabolic activity measured using the MTT assay. This is in line with observations of antiapoptotic treatments, where an improvement in cell membrane conditions appeared before the MTT assay revealed any changes in mitochondrial activity [37,38]. 

IL-1α induced the secretion of IL-6 in accordance with previous findings in brain endothelial cells [39]. The level of IL-6 was reduced by MG-132, a well-known NF-κB inhibitor, with bafilomycin [40]. We have previously shown that hydroquinone-mediated NF-κB inhibition resulted in diminished IL-6 levels, where Resvega returned to the control level independently of NF-κB activation [29]. In the present study, 10 μM Resvega had no additional effect on the IL-6 release upon MG-132 and BafA treatments. Instead, the higher 25 μM concentration increased IL-6 secretion as Resvega did upon hydroquinone exposure. It is possible that Resvega activates the p62–Kelch ECH associating protein 1 (Keap-1)–Nuclear factor erythroid 2-related factor 2 (Nrf2) pathway, whereas in the present study, that pathway was already disrupted and blocked by the proteasome and autophagy inhibitors. The lower 10 μM Resvega concentration was probably not able to induce the p62–Keap-1–Nrf2 pathway, but as shown previously, 25 μM Resvega is able to induce autophagy and could increase IL-6 release through p62–Keap-1–Nrf2 [29,30,41,42,43]. When working properly, p62 induces the release of Nrf2 from its suppressor, Keap-1, and Nrf2 is able to induce the expression of IL-6 [41,44].

In the present study, Resvega prevented the secretion of both IL-1β and IL-8 induced by the dysfunctional intracellular clearance in IL-1α-primed ARPE-19 cells. This is in line with our previous report where we demonstrated that the IL-8 release was secondary to the inflammasome activation and IL-1β secretion [10]. In another study, BafA further enhanced the IL-8 production when added together with IL-1β to RPE cells [45]. Our results do not exclude the possibility that the diminished IL-8 release results from a Resvega-mediated inhibition of the inflammasome. The reduced release of IL-1β and NLRP3, as well as the alleviated caspase-1 activity, support this proposal. In macrophages, resveratrol and omega-3 fatty acids were able to reduce NLRP3 inflammasome activation concurrently with induced autophagy [46,47,48]. Resveratrol and omega-3 fatty acids are known to inhibit NF-κB translocation to the nucleus and diminish inflammasome activation due to the prevention of its priming [32,48]. On the other hand, resveratrol and vitamin C have been shown to reduce ROS production and NLRP3 inflammasome activity in mouse lung tissue and macrophages, respectively [32,49]. The absence of copper has been shown to suppress NLRP3 inflammasome activation in macrophages [50]. In contrast, the depletion of zinc induced NLRP3 activation in macrophages and increased zinc level inhibited inflammasome activation through reduced ROS production in human peritoneal mesothelial cells [51,52].

The IL-1 cytokines are capable of mediating angiogenesis by inducing the production of VEGF, which is also a potential activator of the NLRP3 inflammasome in RPE cells [53,54]. In the present study, VEGF was released after the IL-1α treatment prior to the NLRP3 activation and the secretion of IL-1β. Proteasome and autophagy inhibitors did not exacerbate IL-1α-induced VEGF release but Resvega was able to reduce it to levels even lower than present in untreated control cells. The secretions of IL-1β and VEGF were both reduced in the present study, a finding in accordance with observations that the inhibition of VEGF can inhibit the secretion of IL-1β [55]. Furthermore IL-1β has been observed to induce VEGF secretion, leading to the hypothesis that diminished levels of IL-1β may result in reductions in the release of VEGF [56]. Resveratrol and vitamin C have been shown to reduce VEGF expression in RPE cells [57,58,59]. Ivanescu et al. demonstrated that orally administered Resvega reduced VEGF expression and choroidal neovascularization in a mouse model [60]. Likewise, lutein and zeaxanthin prevented VEGF-mediated neovascularization in human retinal microvascular endothelial cells and diminished VEGF levels in the retina of rats [61,62]. These results suggest that Resvega could have beneficial effects in wet AMD where excessive neovascularization is a problem. However, VEGF is also necessary for RPE cell viability and functionality since it maintains retinal integrity [63]. In addition, RPE-derived VEGF supports choriocapillaris development, survival, and remodeling, which are important for visual function [64,65]. Therefore, the potential antiangiogenic effects of Resvega will need to be taken into account on a case-by-case basis. The local microenvironment that contributes to the VEGF release and neovascularization in AMD contains a plethora of factors, such as proinflammatory cytokines, chemokines, complement factors, macrophages, and microglia cells [66,67]. Additionally, RPE cells release ATP into the subretinal space; this compound is involved in the cooperation between RPE cells with photoreceptors [68]. In human epidermal keratinocyte cells, ATP has been shown to confer protection from oxidative stress by activating the antioxidant defense system [69]. At odds with our findings, it has been reported that extracellular ATP activates the P2X7 receptor, which results in VEGF release [70]. In addition, P2X7 receptor activity maintains a high ATP level and also induces NLRP3 inflammasome activation [26]. We have previously shown that there was an increase in the extracellular levels of ATP following inflammasome activation of cells with disrupted protein clearance [4]. In the present study, Resvega increased the level of extracellular ATP but concurrently decreased VEGF release and NLRP3 inflammasome activation. 

We present evidence that Resvega can reduce NLRP3 inflammasome-mediated inflammation and VEGF production in human RPE cells; these are beneficial responses when searching for options and mechanisms to alleviate RPE cell-related stress.

## 5. Conclusions

Resvega alleviates NLRP3 inflammasome activation in human ARPE-19 cells upon dysfunctional protein clearance. This study warrants further investigations in vitro and in vivo. 

## Data Availability

The data presented in this study are available on request from the corresponding author.

## Supplementary Materials

The following are available online at https://www.mdpi.com/2076-3921/10/1/67/s1, Figure S1: IL-1α-priming did not increase caspase-1 activity in ARPE-19 cells. Caspase-1 activity was measured using a cell-permeable fluorochrome inhibitor of caspase-1 (FLICA, FAM-YVAD-FMK). A green fluorescent signal indicates active caspase-1 attached to the FAM-YVAD-FMK-probe. Nuclei were stained using the blue Hoechst 33342 dye. Pictures were detected using a fluorescent microscope (Zeiss ApoTome.2 Imager M2 microscope). Figure S2: MG-132 (MG; 5 μM) and bafilomycin A1 (BafA; 50 nM) exposure with DMSO control induced caspase-1 activity in IL-1α-primed ARPE-19 cells. Caspase-1 activity was measured using the fluorochrome inhibitor of caspase-1 (FLICA, FAM-YVAD-FMK). A green fluorescent signal indicates active caspase-1 attached to the FAM-YVAD-FMK-probe. Nuclei were stained using the blue Hoechst 33342 dye. Pictures were captured by a fluorescent microscope (Zeiss ApoTome.2 Imager M2 microscope). Figure S3: Resvega (R10μM) decreased MG-132 (MG; 5 μM) and bafilomycin A1 (50 nM; BafA)-induced caspase-1 activity in IL-1α-primed ARPE-19 cells. Caspase-1 activity was measured using the fluorochrome inhibitor of caspase-1 (FLICA, FAM-YVAD-FMK). A green fluorescent signal indicates active caspase-1 attached to the FAM-YVAD-FMK-probe. Nuclei were stained using the blue Hoechst 33342 dye. Pictures were captured by a fluorescent microscope (Zeiss ApoTome.2 Imager M2 microscope).
