# Supplementary material for: Effects of Resvega on Inflammasome Activation in Conjunction with Dysfunctional Intracellular Clearance in Retinal Pigment Epithelial (RPE) Cells
Source: Antioxidants (Basel). 2021 Jan 7;10(1):67. doi: 10.3390/antiox10010067 (PMC7825790; doi:10.3390/antiox10010067)
Supplement: Supplementary file 1 [file antioxidants-10-00067-s001.pdf]

antioxidants

MDPI

Suplemmentary Figures

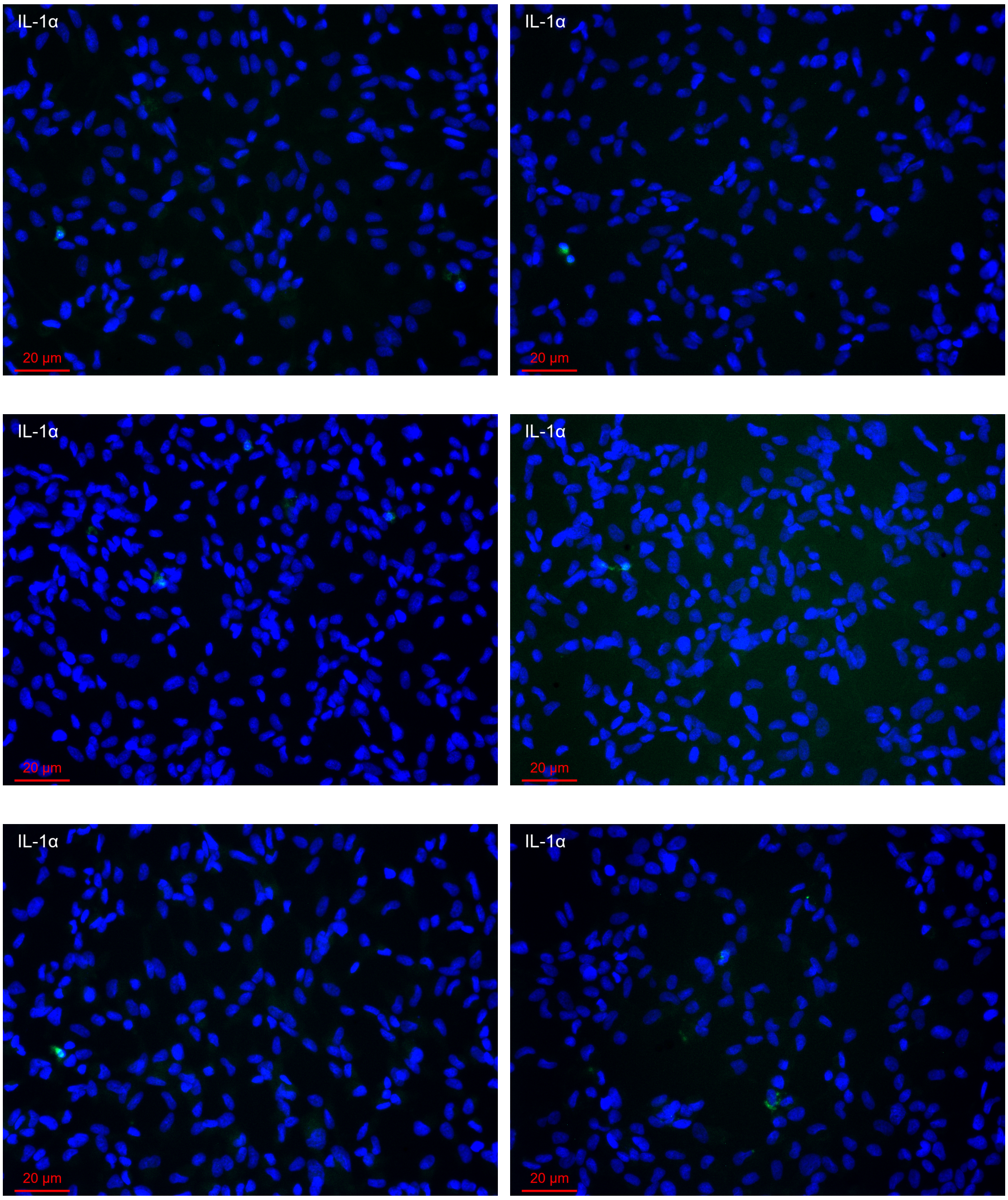

Figure S1. IL-1α-priming did not increase caspase-1 activity in ARPE-19 cells. Caspase-1 activity was measured using a cell-permeable fluorochrome inhibitor of caspase-1 (FLICA, FAM-YVAD-FMK). A green fluorescent signal indicates active caspase-1 attached to the FAM-YVAD-FMK-probe. Nuclei were stained using the blue Hoechst 33342 dye. Pictures were detected using a fluorescent microscope (Zeiss ApoTome.2 Imager M2 microscope).

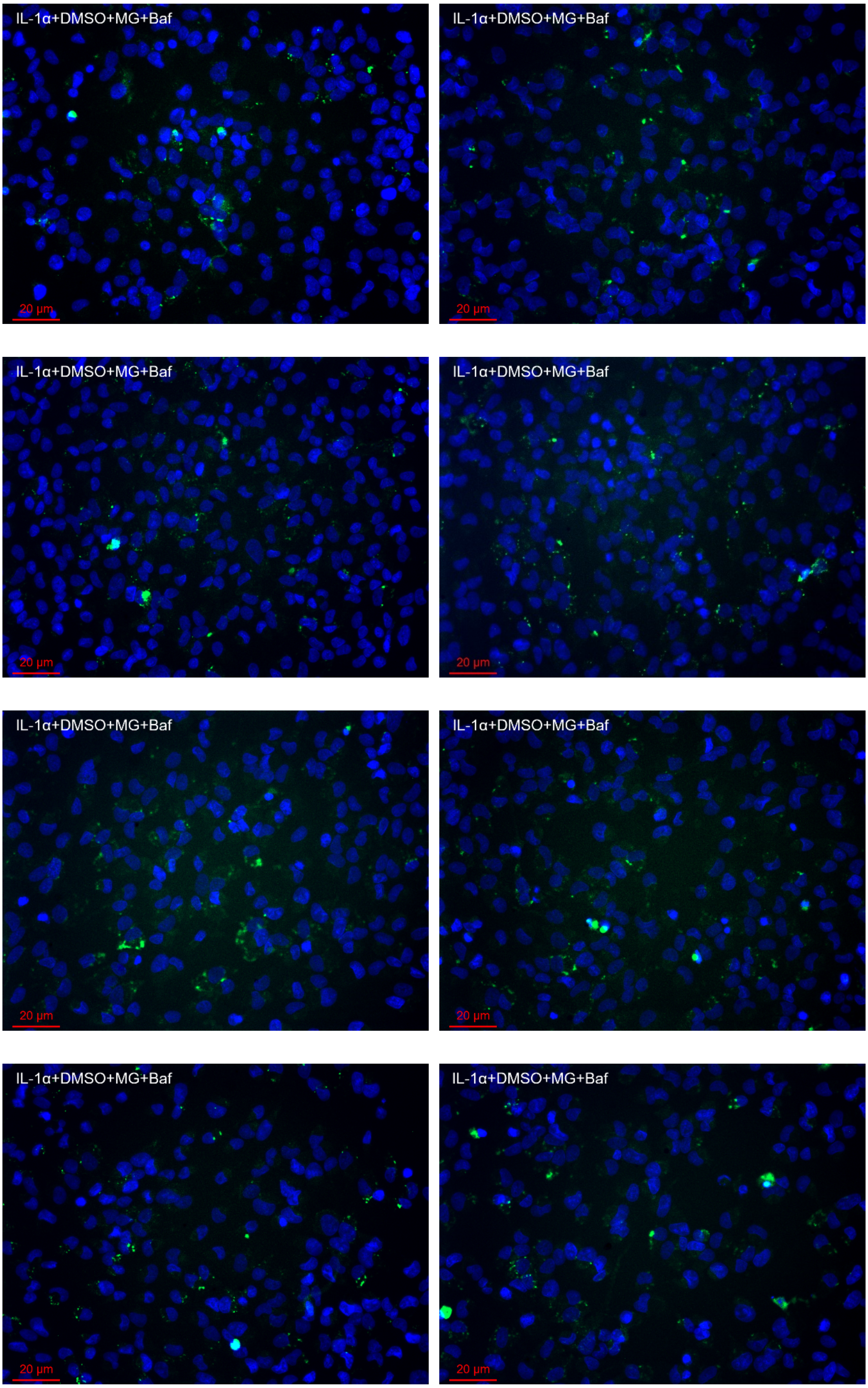

Figure S2. MG-132 (MG; 5 µM) and bafilomycin A1 (BafA; 50 nM) exposure with DMSO control induced caspase-1 activity in IL-1α-primed ARPE-19 cells. Caspase-1 activity was measured using the fluorochrome inhibitor of caspase-1 (FLICA, FAM-YVAD-FMK). A green fluorescent signal indicates active caspase-1 attached to the FAM-YVAD-FMK-probe. Nuclei were stained using the

blue Hoechst 33342 dye. Pictures were captured by a fluorescent microscope (Zeiss ApoTome.2 Imager M2 microscope).

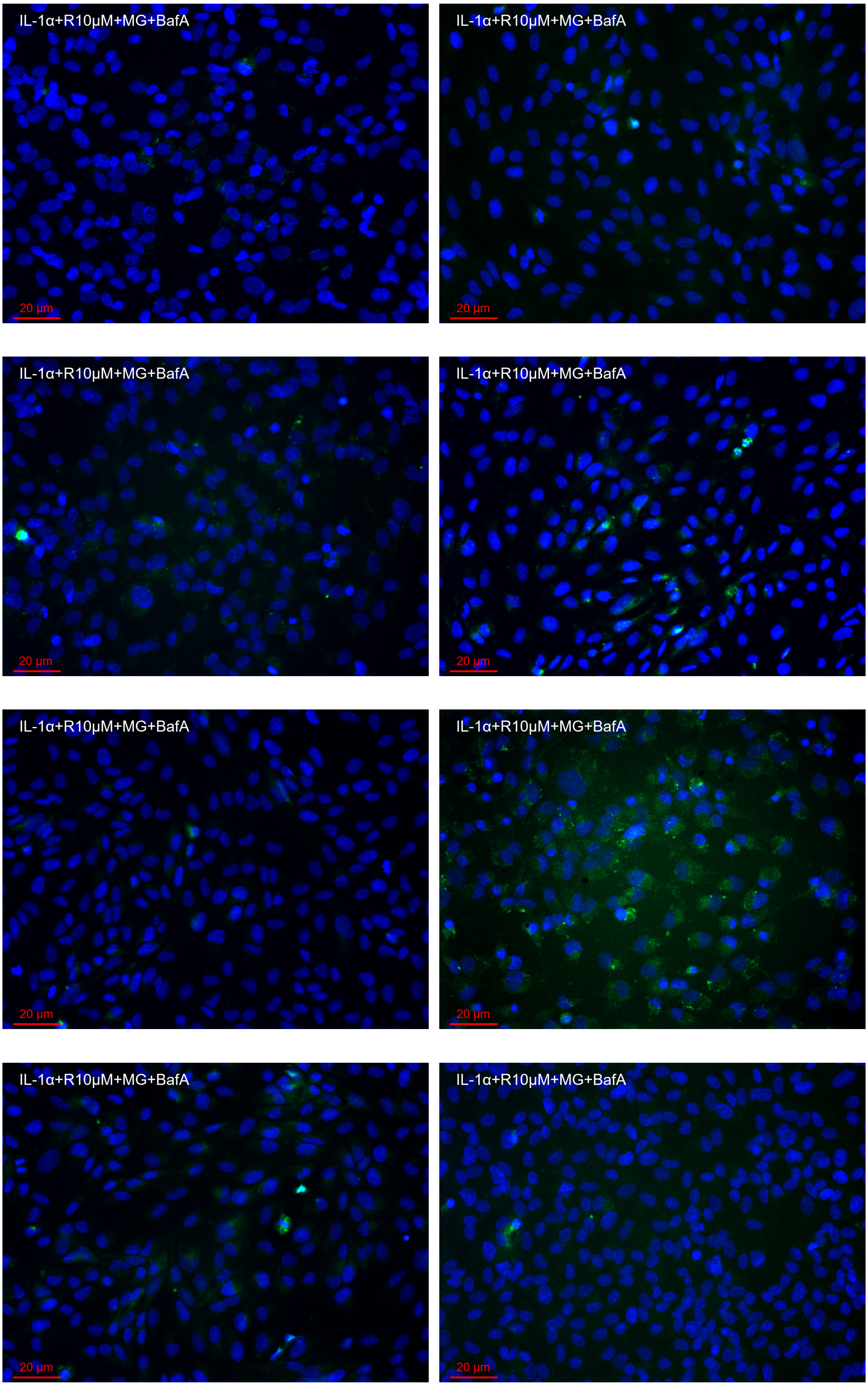

Figure S3. Resvega (R10µM) decreased MG-132 (MG; 5 µM) and bafilomycin A1 (BafA; 50 nM) induced caspase-1 activity in IL-1α-primed ARPE-19 cells. Caspase-1 activity was measured using the fluorochrome inhibitor of caspase-1 (FLICA, FAM-YVAD-FMK). A green fluorescent signal indicates active caspase-1 attached to the FAM-YVAD-FMK-probe. Nuclei were stained using the

blue Hoechst 33342 dye. Pictures were captured by a fluorescent microscope (Zeiss ApoTome.2 Imager M2 microscope).
